# M2‐phenotype tumour‐associated macrophages upregulate the expression of prognostic predictors MMP14 and INHBA in pancreatic cancer

**DOI:** 10.1111/jcmm.17191

**Published:** 2022-02-12

**Authors:** Zhan‐Wen Liang, Jie Yu, Dong‐Mei Gu, Xiao‐Meng Liu, Jin Liu, Meng‐Yao Wu, Meng‐Dan Xu, Meng Shen, Weiming Duan, Wei Li

**Affiliations:** ^1^ Department of Oncology The First Affiliated Hospital of Soochow University Suzhou China; ^2^ Department of Pathology The First Affiliated Hospital of Soochow University Suzhou China

## Abstract

Pancreatic cancer is one of the most lethal gastrointestinal tumours, the most common pathological type is pancreatic adenocarcinoma (PAAD). In recent year, immune imbalanced in tumour microenvironment has been shown to play an important role in the evolution of tumours progression, and the efficacy of immunotherapy has been gradually demonstrated in clinical practice. In this study, we propose to construct an immune‐related prognostic risk model based on immune‐related genes MMP14 and INHBA expression that can assess the prognosis of pancreatic cancer patients and identify potential therapeutic targets for pancreatic cancer, to provide new ideas for the treatment of pancreatic cancer. We also investigate the correlation between macrophage infiltration and MMP14 and INHBA expression. First, the gene expression data of pancreatic cancer and normal pancreatic tissue were obtained from The Cancer Genome Atlas Program (TCGA) and The Genotype‐Tissue Expression public database (GTEx). The differentially expressed immune‐related genes between pancreatic cancer samples and normal sample were screened by R software. Secondly, univariate Cox regression analysis were used to evaluate the relationship between immune‐related genes and the prognosis of pancreatic cancer patients. A polygenic risk score model was constructed by Cox regression analysis. The prognostic nomogram was constructed, and its performance was evaluated comprehensively by internal calibration curve and C‐index. Using the risk model, each patient gets a risk score, and was divided into high‐ or low‐ risk groups. The proportion of 22 types of immune cells infiltration in pancreatic cancer samples was inferred by CIBERSOFT algorithm, correlation analysis (Pearson method) was used to analyse the correlation between the immune‐related genes and immunes cells. Then, we applied macrophage conditioned medium to culture pancreatic cancer cell line PANC1, detected the expression of MMP14 and INHBA by qRT‐PCR and Western blot methods. Knock‐down MMP14 and INHBA in PANC1 cells by transfected with shRNA lentiviruses. Detection of migration ability of pancreatic cells was done by trans‐well cell migration assay. A subcutaneous xenograft tumour model of human pancreatic cancer in nude mice was constructed. In conclusion, an immune‐related gene prognostic model was constructed, patients with high‐risk scores have poorer survival status, M2‐phenotype tumour‐associated macrophages (TAMs) up‐regulate two immune‐related genes, MMP14 and INHBA, which were used to establish the prognostic model. Knock‐down of MMP14 and INHBA inhibited invasion of pancreatic cancer.

## 1 INTRODUCTION

Pancreatic cancer is one of the most lethal solid organ tumour types. As early‐stage pancreatic cancer patients have mild or no clinical symptom, most pancreatic cancer patients are diagnosed at an advanced stage of cancer after obvious symptoms appear or during physical examination and hardly undergo radical resection. Pancreatic cancer patients always result in poor prognosis, with a 5‐year survival rate of <20%.^1^ It is especially important to identify high‐risk pancreatic cancer patients, predict the prognosis of cancer and adjust the treatment option accordingly at the first opportunity.

With the rapid development and cross‐pollination of immunology and oncology in recent decades, a large number of studies have shown that the immune response is closely related to the progression of tumours. One sign of cancer is that tumour cells would adopt the ability to evade the host immune response and maximize their continuous growth potential.^2^ Under normal conditions, tumour cell antigens stimulate an immune response that can lead to the elimination of cancer cells.^3^ In some cases, this response might be inhibited, leading to a suitable immune microenvironment^4^ where inflammatory cells are believed to promote development and tumour formation.^5^, ^6^

Macrophages are the major population of tumour‐associated inflammatory cells and divided into two different phenotypes: macrophage M1 and macrophage M2.^7^ Macrophage M1 cells have anti‐tumour behaviours, while macrophage M2 cells can promote tumour growth, angiogenesis, invasion and metastasis.^8^, ^9^ A previous study illustrated that breast cancer cells separated and progressed into an epithelial‐mesenchymal‐transition (EMT) morphology in the presence of macrophage M2‐conditioned medium (MCM).^9^

Currently, classical TMN stage is the main method to determine the prognosis of cancer; however, due to the high heterogeneity of pancreatic cancer, patients with pancreatic cancer at the same stage often behave differently in terms of tumour recurrence and response to antitumour therapy. Therefore, there is an urgent need to find more reliable parameters to guide prognostic stratification and personalized treatment. With the rapid development of gene expression profiling technology, represented by second‐generation sequencing, we are able to predict patient prognosis by analysing gene expression profiles to further screen for molecular markers with different characteristics. However, since the development of pancreatic cancer is usually a complex process involving multiple genes, a single molecular marker is not sufficient to predict the prognosis of pancreatic cancer patients and guide individualized treatment. Prognostic model constructed based on multiple genes is promising in clinical practice for determining the prognosis of pancreatic cancer patients, and such model can complement the traditional TMN staging method to improve the ability to predict the prognosis. There is no immune‐related marker to systematically assess and predict the prognosis in pancreatic cancer patients before. Here, we used bioinformatic analysis method and experiments to figure out the immune microenvironment feature in pancreatic cancer and built a prognostic model based on immune‐related genes, which could be a tool for predicting survival probabilities of pancreatic cancer patients and investigated the relationship between macrophage and prognosis‐related immune gene expression.

## 2 MATERIALS AND METHODS

### 2.1 Acquisition of data for pancreatic normal samples and tumour samples

The Cancer Genome Atlas (TCGA) data sets and The Genotype‐Tissue Expression (GTEx) data sets were obtained from the UCSC Xena resource (http://xena.ucsc.edu/).^10^ For gene expression, the TCGA RNA‐Seq (polyA + Illumina HiSeq) data were downloaded as log2(norm_count + 1) values, and the GTEx RNA‐Seq data were downloaded as log2(norm_count + 0.001) but was transformed to log2(norm_count + 1) values. Clinical parameters, including patient age, gender, clinic stage and pathological grade, were also acquired. All statistical analyses and bioinformatics analysis were performed using R software (version3.6.0) and its programme package.

### 2.2 Identification of mRNA expressed differentially between pancreatic cancer samples and normal samples

In order to identify differentially expressed genes (DEGs) between pancreatic normal samples and PAAD, we used SVA R package (version: 3.34.0)^11^ to batch the GTEx and TCGA RNA‐Seq and limma R package (version: 3.42.2)^12^ to perform differential expression analysis. The cut‐off value for identifying DEGs was |log2FC (fold‐change) | >1 and *p* < 0.05.

### 2.3 Pathway enrichment and visualization

The gene set enrichment analysis (GSEA) was used to figure out if genes may be statistically critical in the studied gene set.^13^ To investigate the potential function of DEGs in pancreatic samples, we performed pathway enrichment analysis based on GO Biological Process Ontology gene sets with GSEA, to explore the immune function between normal samples and tumour samples and to identify whether the corresponding immune genes were significantly different. Metascape (http://metascape.org) is an online tool that was used for gene enrichment analysis while we tried to compare the different biological pathway in high‐ and low‐risk group of patients, and it also found relevant signalling pathways.^14^

### 2.4 Establishment an immune prognostic model by estimating risk score

Risk scores for each patient were estimated according to genes expression that was multiplied a linear combination of regression coefficient that was acquired from the multivariate Cox regression. Patients then were assigned to high‐risk or low‐risk subgroups based on the most suitable risk score, which were called the cut‐off value; here, the cut‐off value was 2.62. To validate the risk score model, a Kaplan‐Meier OS curve for high‐risk and low‐risk groups was plotted and estimated the area under the curve (AUC) of the receiver that operates the characteristic (ROC) curve.

### 2.5 Validation for prognostic risk prediction and Nomogram development

The nomogram that integrated a variety of immune prognostic model and clinical risk factors was assembled. To assess whether risk score can be used independently to predict prognosis of patients with pancreatic cancer, we performed univariate and multivariate Cox regression analyses on risk score and other clinical pathological factors (eg age and gender), and factors meeting HR >1 or <1 with *p* < 0.05 in both analyses could be used as independent prognostic factors to predict patient's prognosis. To further improve the accuracy of risk score model in predicting patient prognosis and to facilitate clinical use, we established the nomogram. To validate the prognostic risk prediction model, 1 year, 3 years and 5 years OS calibrations were calculated to decide the nomogram model's predictive precision. Decision curve analysis (DCA) was used to compare different models’ advantages.

### 2.6 Estimation of immune cells in pancreatic cancer samples

CIBERSORTx (https://cibersortx.stanford.edu/) is an online tool, which can demonstrate the composition of immune cells in tissue based on an account of gene expression profiles^15^; it has widened gene expression profile database possibilities greatly. We utilized CIBERSORTx to assess 22 types of immune cell including T cells, B cells and macrophages in TCGA data sets. The correlation coefficient and *P* value between risk score, the immune genes and immune infiltrating cells could be calculated by correlation analysis (Pearson method), so as to explore the relationship between immure genes and immune infiltrating cells. Correlation coefficients >0 indicate positive correlation, <0 indicate negative correlation; *p* < 0.05 is considered statistically different.

### 2.7 Cell culture and gene knock‐down techniques

The human monocytic leukaemia cell line THP‐1 and the human pancreatic cancer cell line PANC1 were purchased from the American Type Culture Collection (ATCC). THP‐1 cells were cultured in PRMI‐1640 medium (Gibco). PANC1 cells were cultured in DMEM medium (Gibco). Medium was added with 10% FBS (foetal calf serum; Gibco), 100 mg/ml streptomycin and 100 U/ml penicillin. Cells were cultured at 37°C in a 5% CO_2_ incubator with a humidified atmosphere. The cells were passaged every 2–3 days to sustain growth. The small hairpin RNAs (shRNAs) against human MMP14 or INHBA and the negative control lentiviruses were compounded by Shanghai Taitool Bioscience Co. PANC1 cells are simultaneously transfected with the lentiviruses. After culturing for 72 h, the cells are harvested for analysis.

### 2.8 Cell invasion assay techniques

100 μl of Matrigel (1:30 dilution in serum‐free DMEM medium) was added to the trans‐well polycarbonate filter (Corning) and incubated at 37°C for 6 h. PANC1 cells were washed with PBS and resuspended in DMEM containing 1% FBS at a density of 2 × 10^6^ cells/ml. 600 ml of DMEM medium containing 10% FBS was added to the lower chambers while 100 μl cell suspensions were added to the upper chambers. After 36 h, cell would stain with 1% methylrosanilinium chloride (Beyotime). Cells that had migrated to the underside of the filter were counted in three randomly selected fields.

### 2.9 Macrophage preparation and culture

THP‐1 cells were seeded and cultured in RPMI‐1640 medium supplemented with 200 nM phorbol myristate acetate (PMA) and 10% FBS. After 24 h, medium supplemented with 50 ng/ml human macrophage colony stimulating factor (MCSF, PeproTech Inc.) were used to culture the activated macrophage‐like THP‐1 cells. THP‐1 cells can differentiate into macrophage M2 in 7 days in the presence of MCSF. On Day 7, fresh MCSF‐free medium was used to culture the cells for another 48 h. The culture medium, which was defined as MCM, was then collected and stored in fridge at −80°C.

### 2.10 Real‐time PCR

TRIzol reagent (Invitrogen) was used to separate total RNA according to the manufacturer's protocol. A PrimeScript RT Reagent kit (Takara) was used for reverse transcription and yielded 1 μg total RNA in a final volume of 20 μl, following the protocols of the manufacturer. cDNA corresponding to equal amounts of RNA was used for mRNA quantification by real‐time PCR using the Light Cycler 96 Real‐time Quantitative PCR Detection system (Roche). Reverse and forward primers, corresponding cDNA and SYBR Green PCR master mix (Roche) were contained by the reaction system (13 μl). Using GAPDH gene expression as an internal standard, all data were analysed. The primers for specific genes were as follows: GAPDH, forward, 5′‐GTATCGTGGAAGGACTCATGAC‐3′, reverse, 5′‐ACCACCTTCTTGATGTCATCAT‐3′, product, 280 bp; MMP14, forward, 5′‐CAAGATTGATGCTGCTCTCTTC‐3′, reverse, 5′‐ACTTTGATGTTCTTGGGGTACT‐3′, product, 126 bp; INHBA, forward, 5′‐GGCAAGTTGCTGGATTATAGTG‐3′, reverse, 5′‐CTGAGAGTTGGGTACATCCTTT‐3′, product, 121 bp.

### 2.11 Western blot analysis

Total protein was extracted using a lysis buffer (Cell lysis buffer for Western and IP; P0013; Beyotime). The protein extracts were separated by 10% SDS—polyacrylamide gel electrophoresis (PAGE) and were transferred to nitrocellulose filter (NC) membranes. After 2 h blocking in 5% non‐fat milk, the membranes were incubated overnight at 4°C with rabbit anti‐MMP14 antibody (13130S; Cell Signaling Technology), rabbit anti‐INHBA antibody (ab233083, Abcam) or rabbit anti‐GAPDH antibody (Santa Cruz Biotechnologies). The protein expression was determined using horseradish peroxidase‐conjugated antibodies followed by enhanced chemiluminescence (ECL; Millipore) detection. GAPDH was used as internal control.

### 2.12 Subcutaneous xenograft nude mouse model

Four‐week‐old female BALA/c athymic nude mice (SLAC Laboratory Animal Co. Ltd.) were maintained in a specific room, which is climate‐controlled with foods and water on a 12 h light/dark cycle. PANC1 transfected with negative control, MMP14‐shRNA or INHBA‐shRNA lentiviruses were injected into the subcutaneous of the nude mice in a total volume of 5 × 10^6^ cells. At the end of the experiment, the mice were anaesthetized. The tumours were resected and formalin‐fixed.

## 3 RESULTS

### 3.1 Stromal landscape and immune landscape are different between pancreatic normal sample and PAAD

**FIGURE 1:**
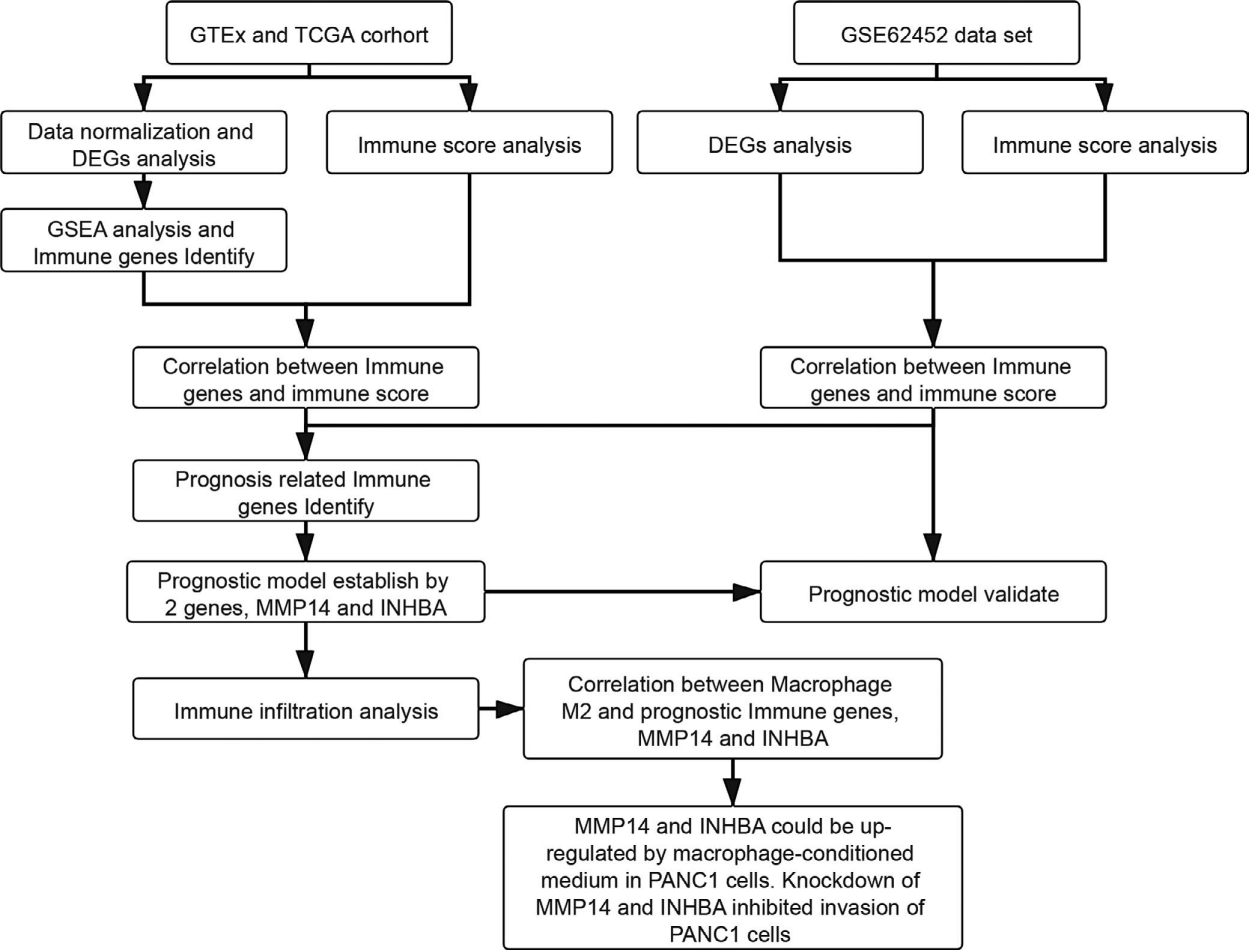
Workflow chart of this study

**FIGURE 2:**
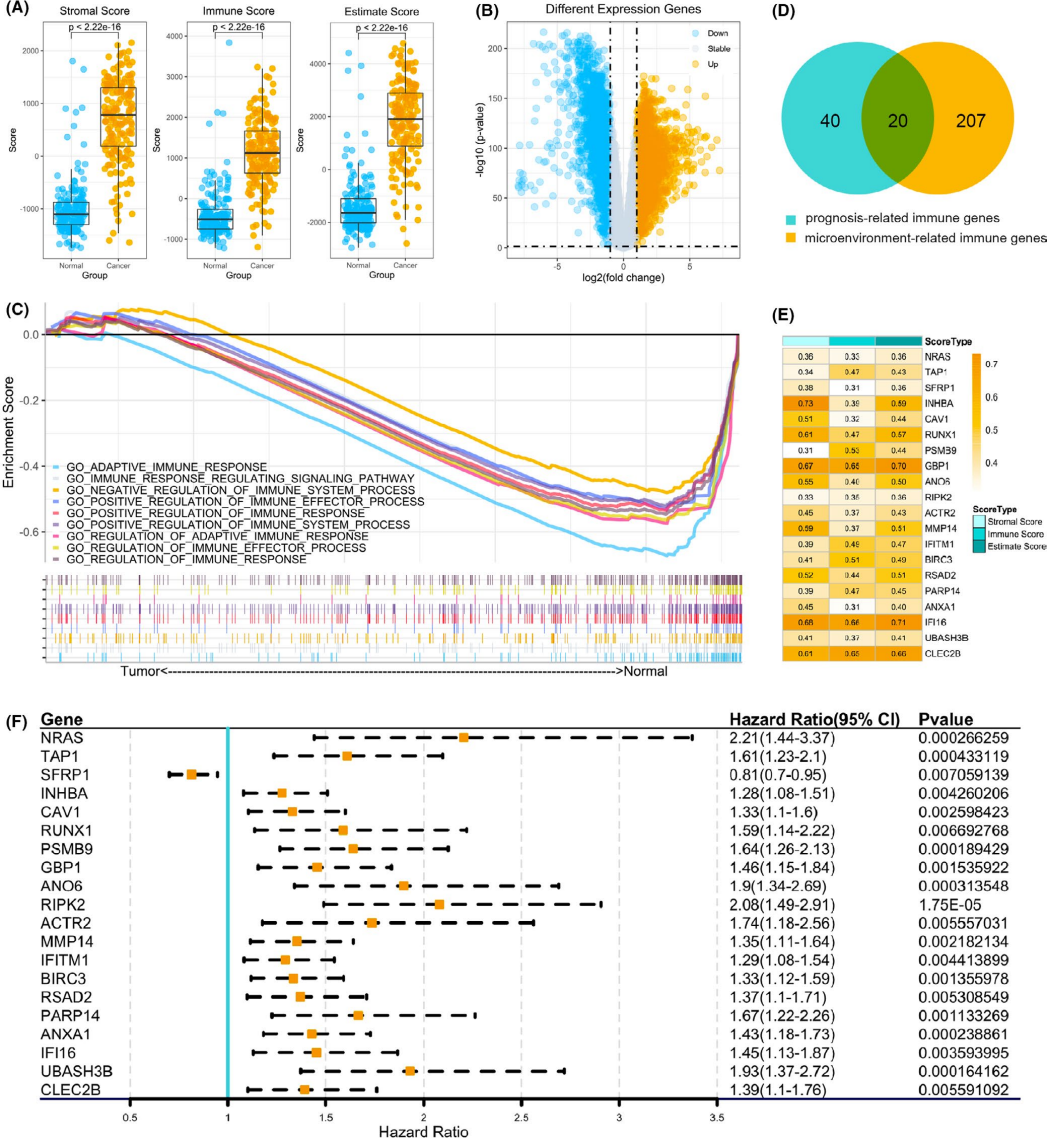
Analysis of the tumour microenvironment and the prognostic immune genes between pancreatic normal samples and tumour samples. (A) Stromal scores, immune scores and estimate scores between pancreatic normal samples and cancer samples. (B) The differentially expressed genes (DEGs) between pancreatic cancer and normal samples. (C) Geneset enrichment analysis of DEGs. (D) A Venn diagram for prognosis‐related genes and tumour microenvironment related genes. (E) Pearson correlation coefficient between 20 prognosis‐related immune genes and microenvironment scores. (F) Univariate Cox regression of 20 prognosis‐related immune genes

The flowchart of this study is shown in Figure 1. Gene expression profiles of 167 pancreatic normal samples from the GTEx database, 4 pancreatic normal samples from TCGA database, in addition to 177 pancreatic tumour samples from the TCGA database, were combined. According to the ESTIMATE algorithm,^16^ the immune scores, estimate scores and stromal scores of these combined 348 samples were estimated based on their respective RNA expression profiles (Figure 2A). The difference in scores between normal samples and PAAD is large, which showed that the microenvironment landscape in cancer samples is different than normal samples.

### 3.2 A total 4620 genes were differentially expressed between normal pancreatic samples and PAAD

The genome wide mRNA expression profiles in pancreatic normal samples and tumour samples were combined from GTEx and TCGA databases (including 171 pancreatic normal samples and 177 tumour samples). |log2 FC (fold‐change) | >1 and *p* < 0.05 were the cut‐off criteria. Compared with normal pancreatic samples, we identified 2304 and 2316 genes that were significantly up‐ and down‐regulated respectively. The distribution of differentially expressed genes (DEGs) between normal samples and pancreatic cancer samples is shown by volcano plot (Figure 2B).

### 3.3 A weakened immune phenotype in pancreatic cancer

Considering the different immune landscape between normal pancreatic samples and PAAD samples, we evaluated the distinct immune biological processes and genes between PAAD and normal pancreatic samples, RNA‐seq data were utilized for all 4,620 DEGs from the 348 pancreatic samples. Gene set enrichment analysis (GSEA) showed pancreatic cancers were negatively relevant to nine immune biological processes, specifically showing a different immune microenvironment property between pancreatic normal samples and cancer samples, indicating a weakened microenvironment immune response in PAAD (Figure 2C) (Table S1). In these 9‐biological immune‐related processes, 487 immune‐related genes were obtained from the 4,620 DEGs (Table S2). To further verify the association between these 487 immune‐related genes and the prognosis of patients with pancreatic cancer, univariate Cox regression was used to analyse the 487 immune‐related genes in the TCGA data sets, the results showed that 60 of the immune‐related genes met the condition of HR >1 or <1 with *p* < 0.01, confirming that these 60 immune‐related genes were indeed strongly associated with the prognosis of patients with pancreatic cancer (Table S3). Furthermore, correlation analysis (Pearson method) was used to figure out the correlation between these 487 immune‐related genes and microenvironment landscape scores. With the condition of |*R*| >0.3 and *p* < 0.01, 227 immune‐related genes were extracted; and then, Venn plot was used to get genes related to prognosis and the microenvironment landscape (Figure 2D). Indeed, 20 genes were identified, and their Pearson correlation coefficients with microenvironment landscape were shown in Figure 2E, the prognostic hazard ratio (HR) indexes of these 20 immune‐related genes are shown in Figure 2F.

### 3.4 An immune prognostic model based on MMP14 and INHBA expression was established and evaluated in TCGA data set

**FIGURE 3:**
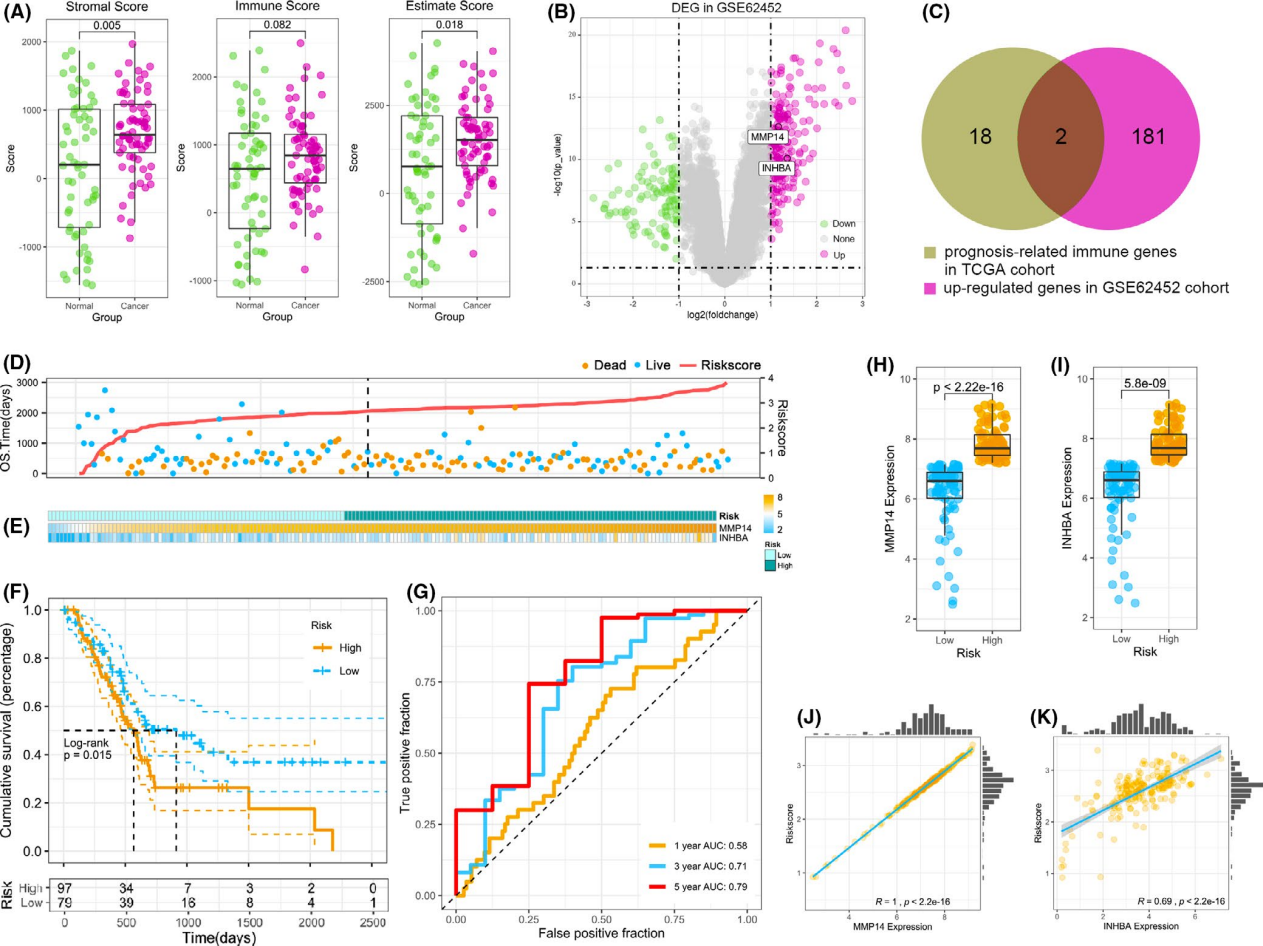
Analysis of the survival of pancreatic cancer patients in The Cancer Genome Atlas Program (TCGA) training data set. (A) Stromal scores, immune scores and estimate scores between pancreatic normal samples and cancer samples in the GEO cohort. (B) The differentially expressed genes (DEGs) between normal samples and pancreatic cancer samples in the GEO cohort. (C) Venn diagram for prognosis‐related immune genes in The Cancer Genome Atlas Program (TCGA) cohort and up‐regulated genes in the GSE62452 cohort. (D) Patients with high‐risk scores correlated with shorter survival time and a higher death rate in pancreatic cancer. (E) The distribution of pancreatic cancer patients’ risk scores and the expression of MMP14 and INHBA. (F) Kaplan‐Meier analysis for the prognostic model. (G) The immune prognostic evaluation model. (H) MMP14 gene expression between the low‐risk and high‐risk groups. (I) INHBA gene expression between the low‐risk and high‐risk groups. (J) Correlation analysis between MMP14 gene expression and risk scores. (K) Correlation analysis between INHBA gene expression and risk scores

To confirm the robust of these 20 immune‐related genes were indeed strongly associated with the prognosis of patients with pancreatic cancer, the GEO data set GSE62452 was utilized for further validation analysis. We first calculated stromal scores, immune scores and estimate scores for GEO data set GSE62452 (Figure 3A). Like the TCGA cohort, scores of cancerous microenvironments landscape were different from normal samples. We also analysed differentially expressed genes (DEGs) between pancreatic normal samples and tumorous samples in the GSE62452 cohort (Figure 3B). Next, we used a Venn plot to identify up‐regulated genes in the GEO cohort and 20 prognosis‐immune‐related genes obtained from the TCGA cohort (Figure 3C) to confirm which immune‐related genes were strongly associated with prognosis; only two genes were identified, MMP14 and INHBA. To establish immune‐related gene prognostic model, multivariate Cox regression analysis was executed to establish a prognostic model for pancreatic cancer patients based on gene expression. That is, risk scores = (0.3732 × MMP14 expression) + (− 0.0212 × INHBA expression). With this model, we estimated risk scores for patients. To investigate whether risk score is a reliable predictor of prognosis in patients with pancreatic cancer, we divided patients with pancreatic cancer into high‐ and low‐risk as stated by the optimum risk score cut‐off value 2.62. Figure 3F presents the results of Kaplan‐Meier survival analysis of the two groups of patients with pancreatic cancer in TCGA data set, and the prognosis of patients with pancreatic cancer was worse in the high‐risk group compared with the low‐risk group (*p* < 0.05). Figure 3D sorts the patients into high‐ and low‐ risk groups based on the cut‐off value (2.62) by ranking them from low‐ to high‐risk scores and depicts the difference in survival time and outcome between patients in the high‐ and low‐risk group, and it is clear from the figure that patients in the high‐risk group have a higher mortality rate compared with patients in the low‐risk group. The two immune‐related gene expression levels as well as the distribution of risk groups were also analysed for each patient, MMP14 and INHBA expression levels were significantly different between the low‐risk and the high‐risk subgroups (Figure 3E). Specifically, in the TCGA training data set, MMP14 and INHBA expression levels were very low among the low‐risk group compared with high‐risk group (Figure 3H,I). MMP14 and INHBA expression levels were positively correlated with risk scores, the Pearson correlation coefficients were 1 and 0.69 respectively (Figure 3J,K). The ROC curve was plotted to further evaluate the accuracy of the predictive ability of the immune prognostic model, and the larger area under the curve (AUC), the better the predictive ability of the model. AUC of the prognostic model at 1 year, 3 years and 5 years was 0.58, 0.71 and 0.79, respectively, as demonstrated by the receiver operating characteristic (ROC) curves (Figure 3G), which illustrates the model is sensitive and specific to forecasting pancreatic cancer patient prognosis.

### 3.5 The immune prognostic model has the ability to accurately predict the prognosis of patients with pancreatic cancer in GEO data set

**FIGURE 4:**
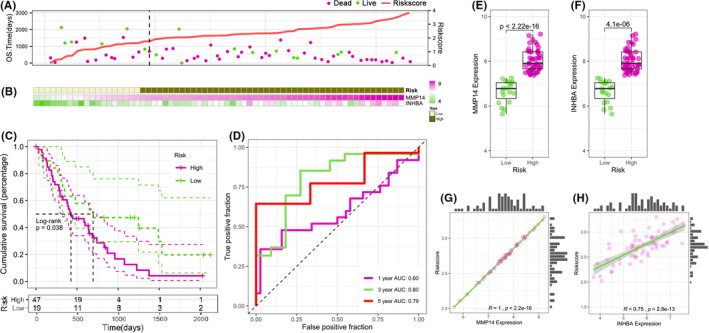
Analysis of the survival of pancreatic cancer patients in the GEO validation cohort. (A) Patients with high‐risk scores correlated with shorter survival time and a higher death rate from pancreatic cancer. (B) A heatmap for pancreatic cancer patient's risk score and gene expression of MMP14 or INHBA. (C) Kaplan‐Meier analysis for the prognostic model. (D) The immune prognostic evaluation model. (E) Comparison of MMP14 gene expression in the high‐risk group and low‐risk group. (F) Comparison of INHBA gene expression in the high‐risk group and low‐risk group. (G) Correlation analysis between MMP14 gene expression and risk scores. (H) Correlation analysis between INHBA gene expression and risk scores

We utilized the GEO data set GSE62452 for validation and application of our immune prognostic model. Using the same method and cut‐off value to calculate risk value, patients were categorized as high‐risk or low‐risk. The K‐M survival curve was used to assess the ability of the risk score model to predict the prognosis of pancreatic cancer patients in GEO data set, and the results show that the overall survival of patients in the high‐risk group was significantly shorter (*p* = 0.038) as shown in Figure 4C. Figure 4A sorts the patients into high‐ and low‐risk groups based on the cut‐off value (2.62) by ranking them from low‐ to high‐risk scores and depicts the difference in survival time and outcome between patients in the high‐ and low‐risk groups, and it is clear from the figure that patients in the high‐risk group have a higher mortality rate compared with patients in the low‐risk group. The two immune‐related gene expression levels as well as the distribution of risk groups were also analysed for each patient, MMP14 and INHBA expression levels were correlated with risk scores in GEO validation data set (Figure 4B). The expression level of MMP14 and INHBA was very low in the low‐risk group compared with high‐risk group (Figure 4E,F). MMP14 and INHBA expression levels were positively correlated with risk scores, the Pearson correlation coefficients were 1 and 0.75 respectively (Figure 4G,H). The ROC curve was plotted to further evaluate the accuracy of the predictive ability of the immune prognostic model, the results show that the area under the curve of the model at 1 year, 3 years and 5 years was 0.60, 0.80 and 0.79 respectively (Figure 4D). This result revealed the prognostic model could predict survival risk sensitivity and specificity.

### 3.6 A nomogram was developed and could facilitate the use the immune prognostic model

**FIGURE 5:**
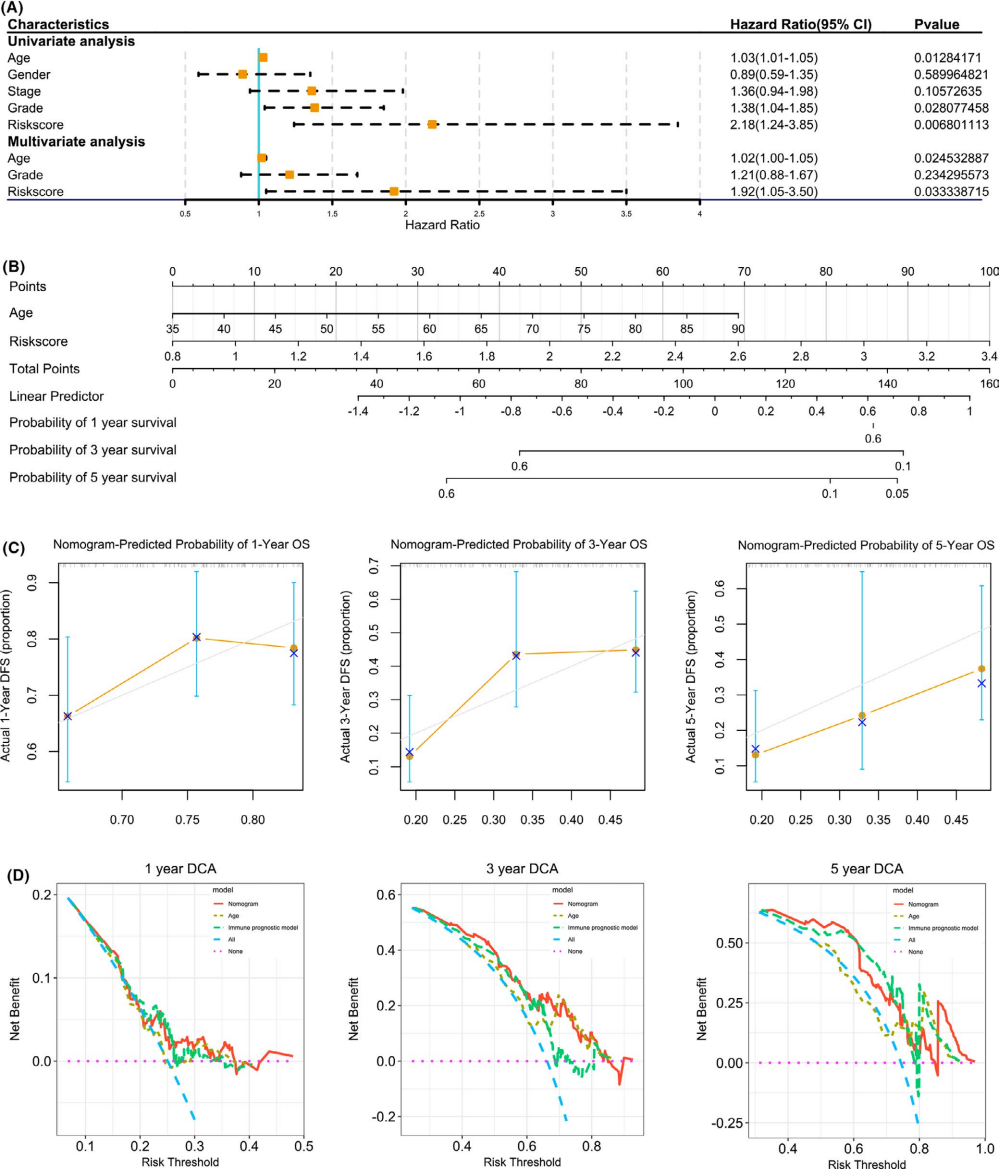
Nomogram forecasting overall survival for pancreatic patients. (A) Forrest plot of the univariate and multivariate Cox regression analysis in pancreatic cancer. (B) Nomogram for risk scores and patient 1 year, 3 years and 5 years survival. (C) The calibration plot for internal validation of the nomogram. (D) The decision curve analysis (DCA) curves of age, and immune prognostic model were smaller than calculated with the nomogram in calculated net benefit

To investigate whether risk score could independently predict patient prognosis, we performed independent prognostic analysis on risk score and other clinical pathological factors in TCGA data set including age, gender, stage and grade. Univariate Cox regression analysis indicated that age (*p* < 0.01), grade (*p* < 0.05) and risk score (*p* < 0.01) calculated from the two immune‐related genes were independent prognostic factors for OS. While multivariate Cox regression analysis indicated that age (*p* < 0.05) and risk score (*p* < 0.05) were independent prognostic factors for OS (Figure 5A). In order to predict the prognosis of pancreatic cancer patients more conveniently and accurately, a nomogram was then constructed to predict 1 year, 3 years and 5 years OS based on two independent prognostic factors analysed by multivariate Cox regression including age and risk score. The first row is the score scale of individual factors, the second and the third rows are the age and risk score. Accordingly, the scores corresponding to the ages and risk scores are derived, and the total scores obtained are added and compared with the total score scale in the fourth row to derive the 1 year, 3 years and 5 years survival rates of patients and to visually analyse the prognosis of pancreatic patients (Figure 5B). The nomogram was suitable to estimate the mortality showed by the calibration plots (Figure 5C). Decision curve analysis showed that the nomogram demonstrated the most excellent net benefit for 1 year, 3 years and 5 years OS (Figure 5D), further shows that the nomogram has high predictive consistency.

### 3.7 The risk score could partially reflect the state of the immune microenvironment

**FIGURE 6:**
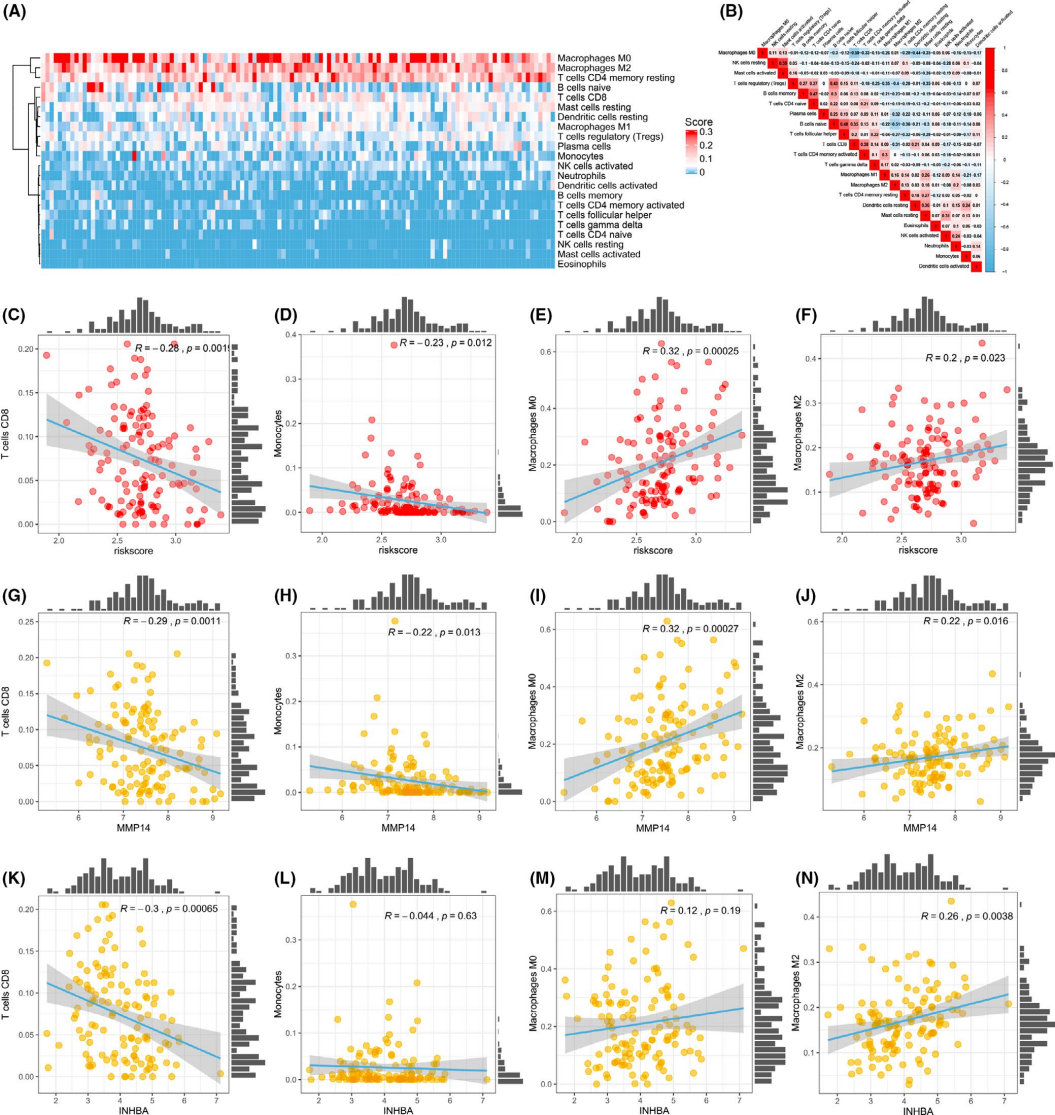
Correlation of immune cells and genes in The Cancer Genome Atlas Program (TCGA) training data set. (A) Immune cell infiltration in pancreatic cancer patients. (B) Pearson correlation coefficient between the 22 immune cells in pancreatic cancer cells. (C‐F) Correlation analyses between risk scores and immune cells. (G‐J) Correlation analysis between MMP14 gene expression and immune cells (T cells CD8, T cells CD4 memory activated, neutrophils and macrophage M2). (K‐N) Correlation analysis between INHBA gene expression and immune cells (T cells CD8, T cells CD4 memory activated, neutrophils and macrophage M2)

The immune prognostic risk model is composed of immune‐related genes, we hypothesized that the risk score could partially reflect the state of the immune microenvironment. First, CIBERSORTx was used to evaluate 22 immune cells proportions, Figure 6A,B demonstrates the composition of immune cells in pancreatic cancer samples from the TCGA pancreatic cancer data set. Then, the relationship between MMP14 or INHBA and immune cells was analysed. With *p* < 0.05 and correlation coefficient >0.2 or <−0.2, the immune cells were determined to be the property relating to the risk scores in the TCGA data set (Figure 6C‐F). From this, MMP14 was related to four different immune cells (T cells CD8, T cells CD4 memory activated, neutrophils and macrophage M2; Figure 6G‐J) and INHBA related to the same immune cells (Figure 6K‐N).

### 3.8 Tumour immune landscape and the immune checkpoints analysis

**FIGURE 7:**
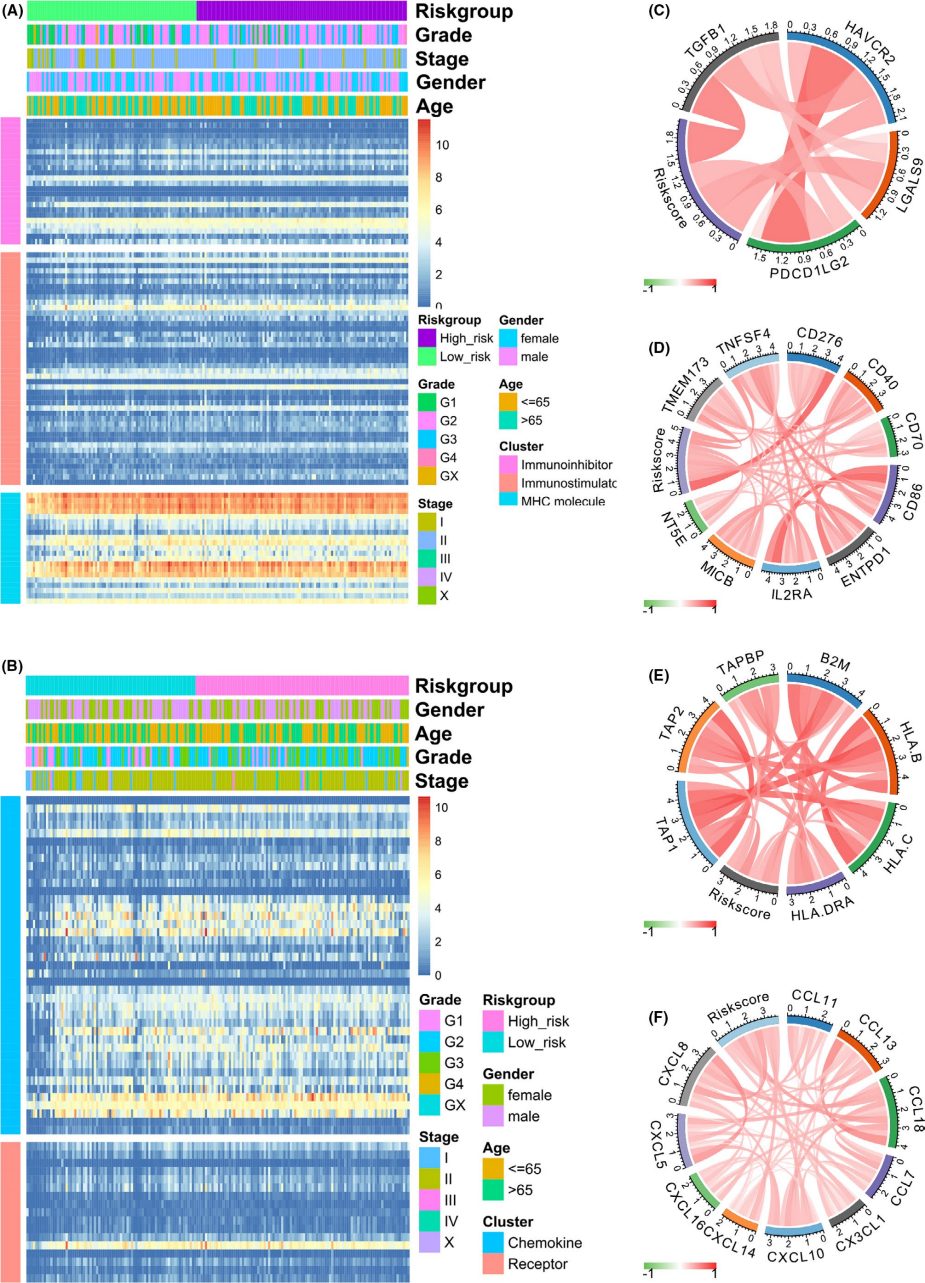
Tumour immune landscape and chemokines association with risk score. (A) Association between immunomodulator and risk score. (B) Association between chemokine and risk score. (C) The expressions of immunoinhibitor were positively correlated to the risk score. (D) The expressions of immunostimulator were positively correlated to the risk score. (E) The expressions of MHC molecules were positively correlated to the risk score. (F) The expressions of chemokines were positively correlated to the risk score

In order to explore the correlation between the immune response and risk score based on immure‐related genes, immunomodulator including immune inhibitor, immune stimulator and MHC molecule (Figure 7A), chemokine including chemokine and receptor (Figure 7B) were taken into analysation. We discovered that the immune landscape between high‐risk and low‐risk groups patients was apparently different. Modulator and immune checkpoints have been the subject of a wave of new studies in immune regulation, and strategies are being promised by immune checkpoint blockade therapies in cancer's treatment. To further explore which immune molecule was correlated with risk score, we performed correlation analysis and found that the immune inhibitor including TGFB1, HAVCR2, LGALS9 and PDCD1G2 was positively correlated with risk score (Figure 7C). The immune stimulator including TNFSF4, CD276, CD40, CD70, CD86, ENTPD1, IL2RA, MICB, NT5E and TMEM173 was positively correlated with risk score (Figure 7D). The MHC molecule including TAPBP, B2 M, HLA.B, HLA.C, HLA.DRA, TAP1 and TAP2 was positively correlated with risk score (Figure 7E). The chemokine including CCL11, CCL13, CCL18, CCL7, CX3CL1, CXCL10, CXCL14, CXCL16, CXCL5 and CXCL8 was positively correlated with risk score (Figure 7F).

### 3.9 Macrophage M2 could upregulate the prognostic genes MMP14 and INHBA in pancreatic cancer

**FIGURE 8:**
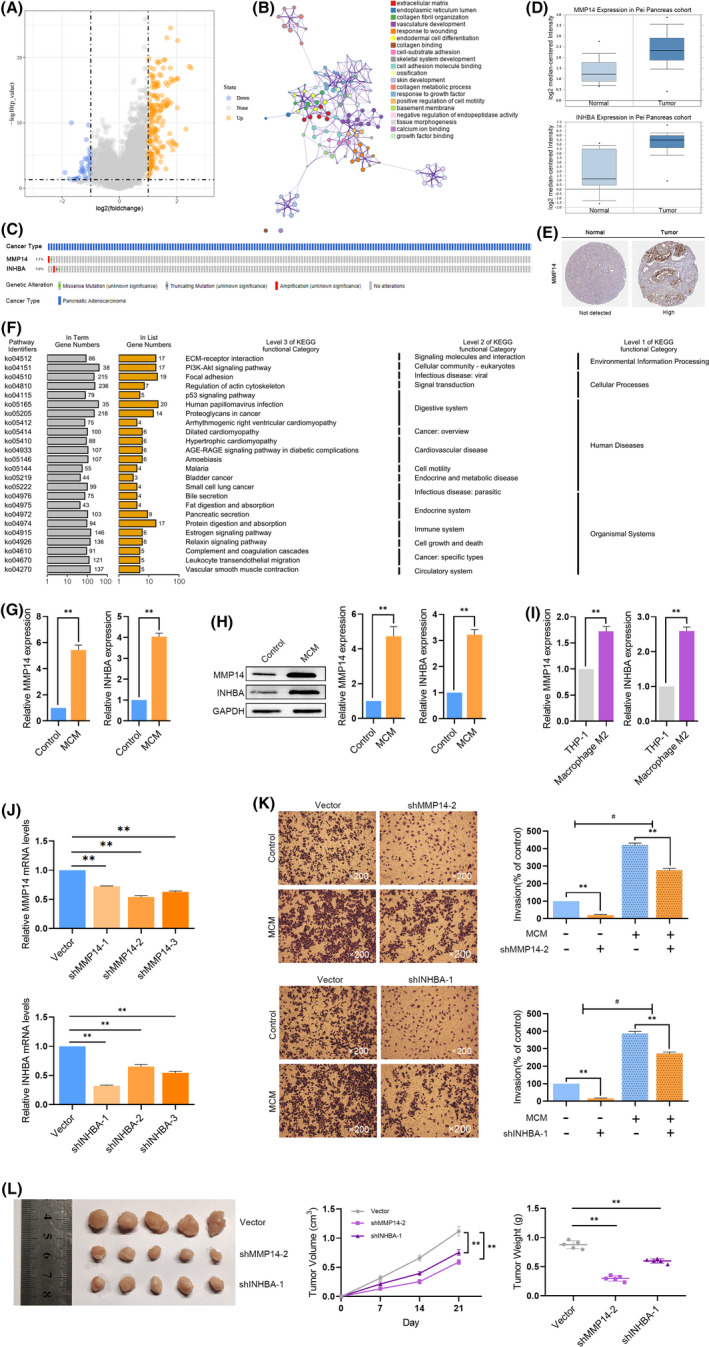
Up‐regulation of MMP14 and INHBA by M2‐phenotype macrophage in pancreatic cancer. (A) The differentially expressed genes (DEGs) between high‐risk group and low‐risk group patients. (B) Functional analysis of DEGs. (C) The expression alteration profiles of MMP14 and INHBA in The Cancer Genome Atlas Program (TCGA) pancreatic cohort. (D) The expression profiles of MMP14 and INHBA in the Oncomine Pei pancreas cohort. (E) Human Protein Atlas dataset showed the protein expression of MMP14 in pancreatic cancer and normal samples. (F) Encyclopedia of Genes and Genomes (KEGG) pathway enrichment of DEGs. (G) qPCR showed the expression of MMP14 and INHBA is up‐regulated by MCM. **p < 0.01 (H) The protein expression analysis of MMP14 and INHBA in PANC1 cell lines treated with MCM. **p < 0.01 (I) The expression of MMP14 and INHBA rises when THP‐1 cells are polarized to M2‐phenotype macrophages. **p < 0.01 (J) MMP14‐shRNA decreased MMP14 expression, INHBA‐shRNA decreased INHBA expression. **p < 0.01 (K) MMP14‐shRNA and INHBA‐shRNA inhibited invasion of pancreatic cancer cells while M2‐conditioned medium (MCM) could weaken the phenomenon. **p < 0.01 (L) Subcutaneous xenograft nude mouse models confirmed that the growth of tumours was weakened by knocking down the expression of MMP14 and INHBA. **p < 0.01

To investigate the different clinicopathological features of the different risk groups of the pancreatic cancer, the pathway was identified by us underlying the risk score, differential expression analysis was performed and got 251 DEGs between the high‐risk group and low‐risk group (|log FC| > 1, *p* < 0.05) (Figure 8A). We used Metascape to annotate the function of DEGs and charted the top 20 significant biological processes, as shown in Figure 8B. GO analysis of the DEGs revealed that ‘extracellular matrix organization’ were enriched in biological processes and pathways, which might be correlated with pancreatic cancer's malignant progression. The ‘extracellular matrix organization’ is the most significant biological process, which reveals that in the high‐risk subgroup patients, cancer cells may have more progression ability than the low‐risk subgroup. Furthermore, only 1.1% patients were containing genetic alterations in MMP14 and 1.6% genetic alterations in INHBA (Figure 8C) shown in cBioportal for TCGA database, demonstrated that these two genes contained only little genetic alteration. Moreover, the mRNA expression of MMP14 and INHBA was significantly up‐regulated compared with non‐tumour samples in the Oncomine Pei pancreas cohort (Figure 8D). MMP14's protein expression was further explored by us in the human protein profiles and shown in Figure 8E. However, we did not find INHBA protein expression in the database.

We then used Metascape to annotate the function of DEGs through KEGG analyse, analysis of the DEGs revealed that ‘ECM receptor interaction’ was the most significant pathway, which might be highly correlated with malignant progression of pancreatic cancer. These results could suggest that MMP14 and INHBA up‐regulated in tumour samples not by gene‐alteration. With the high‐risk score counted with the expression of MMP14 and INHBA, patients might suffer more malignant progression.

We detected the expression levels of MMP14 and INHBA in PANC1 cells upon treatment with M2‐phenotype macrophage conditioned medium (MCM) and found these two immune prognostic genes could be up‐regulated by M2‐phenotype macrophage (Figure 8G). Furthermore, MMP14 and INHBA protein were up‐regulated as well (Figure 8H). Indeed, MMP14 and INHBA were significantly up‐regulated upon treatments with MCM. The expression of MMP14 and INHBA also rises when THP‐1 cells are polarized to M2‐phenotype macrophages (Figure 8I). To validate in the high‐risk group patients, cancer cells may have the more progression ability than the low‐risk group, we performed trans‐well assay by controlled the risk scores parameter MMP14 and INHBA. The effects of MCM on the invasion of pancreatic cancer cells were also evaluated by trans‐well assay. PANC1 cell lines were transfected with MMP14‐shRNA (sh‐control, sh‐1, sh‐2 and sh‐3) or INHBA‐shRNA (sh‐control, sh‐1, sh‐2 and sh‐3), respectively, which resulted in decreased of MMP14 or INHBA (Figure 8J). We then used the lowest expression of MMP14 or INHBA cell lines, MMP14 sh‐2 and INHBA sh‐1 for follow‐up experiments, as shown in Figure 8K, treatment with MCM increased the invasive ability of PANC1 cells. Knockdown immune‐related genes MMP14 or INHBA decreased the invasion of pancreatic cancer cells, which could also be promoted by MCM. Subcutaneous xenograft nude mouse models confirmed that the growth of tumours was weakened by knocking down the expression of MMP14 and INHBA (Figure 8L).

## 4 DISCUSSION

Although the treatment of pancreatic cancer has made great progress, the incidence and mortality rate of pancreatic cancer are still in the forefront and on the rise.^17^, ^18^ The prognosis of pancreatic cancer is relatively poor worldwide, which leads to a fairly similar morbidity and mortality rate.^19^ The number of pancreatic cancer patients in China is a major economic and social burden.^20^

Tumours are highly heterogeneous, which prose a great challenge for patient prognosis prediction and individualized treatment. Therefore, there is an urgent need to find new markers to provide new ideas for the classification and treatment of pancreatic cancer and improve patient prognosis. Currently, several research teams have constructed prognostic risk models based on different molecular characteristics such as metabolism,^21^ cell cycle^22^ and chemokines receptors^23^ in order to predict the efficacy of treatment and the prognosis of cancer patients. Since tumour evolution is a complex process, such multigene‐based prognostic risk models are more accurate in prediction the prognosis of cancer patients than single gene prediction. Currently, there is no report on the construction of prognostic risk models based on immune‐related genes to predict the prognosis of patients with pancreatic cancer.

We first screened differentially expressed immune‐related genes in pancreatic cancer by bioinformatics analysis in different database, the immune‐related genes essentially associated with prognosis were further examined. Next, the immune‐related prognosis model was established by the expression of immune‐related genes MMP14 and INHBA in pancreatic cancer. Gene Expression Omnibus (GEO) data set was used to validate the prognostic model. To play molecular and clinical characteristics complementary value, the immune prognosis model was then composed with clinical characteristics for constructing nomogram, which improved the prognosis estimation of pancreatic cancer. Furthermore, CIBERSORTx was used to evaluate 22 immune cell relative proportions, high numbers of neutrophils and macrophage M2, combined with low numbers of activated CD4+ memory T cells and CD8+ T cells were infiltrating in pancreatic cancer patients. The two immune‐related genes, MMP14 and INHBA, used to construct the prognostic model were positively correlated with the degree of macrophage M2 infiltration in the TCGA data sets. Bioprocess analysis revealed that the ‘extracellular matrix organization’ is the most significant biological process between high‐risk and low‐risk subgroup patients, showed cancer cells may have stronger progressive ability in high‐risk than the low‐risk group. Treating pancreatic cancer cells PANC1 with macrophage M2‐conditioned medium lead to up‐regulated MMP14 and INHBA expression, suggesting macrophages M2 up‐regulate these genes and can be used as risk predictors. Effects of MCM and the immune genes MMP14, INHBA on the invasion of pancreatic cancer cells were evaluated by trans‐well assay. Knock‐down of MMP14 and INHBA inhibited invasion of PANC1 cells. In addition, subcutaneous xenograft nude mouse models confirmed that the growth of tumours was weakened by knock‐down MMP14 and INHBA. Therefore, macrophage M2 cells may support cancer progression and tumour growth.^24^, ^25^

Matrix metalloproteinases (MMP) are a category of enzymes mediating alterations in the TME that occurs throughout progression and tumour development.^26^, ^27^ In humans, 6 distinct membrane‐type MMPs (MT‐MMPs) have been discovered. MMP14 was first depicted by Sato et al.^28^, ^29^ as a transmembrane protein activating pro‐MMP2 to induce tumour cell invasion.^30^, ^31^ In several cancer types, MMP14 is up‐regulated and contributes to advancing inflammation, angiogenesis, metastasis and cancer cell invasion. MMP14's potential clinical relevance in pancreatic cancer has been addressed applying patient‐derived tumour substance.^32^, ^33^, ^34^, ^35^ A rather consistent picture was shown by studies with respect to MMP14 overexpression in tumours compared with control sections.^36^ Tumour‐associated macrophages M2 expressing MMP14 are involved in complicated matrix remodelling, as demonstrated in a colorectal cancer orthotopic mouse model.^37^ We discovered that MMP14 expression is higher in pancreatic cancer samples compared with corresponding normal samples, and the^38^ MMP14 expression is correlated with macrophage M2.

INHBA, which encodes a member of the TGF‐β (transforming growth factor‐beta) superfamily of proteins, has been discovered to play a vital function in distinct kinds of cancer.^39^, ^40^ Okano et al. reported that the INHBA gene's low expression was related to substantially better 5‐year OS in colorectal cancer patients.^41^, ^42^, ^43^ Oshima et al. discovered that gastric cancer patients with low‐INHBA expression had substantially better OS compared with those with high‐INHBA expression.^39^, ^44^ Other independent laboratories demonstrated that INHBA expression could be a utilitarian method to forecast gastric cancer prognosis.^45^, ^46^ We discovered that INHBA was expressed higher in pancreatic cancer samples and correlated with macrophage M2 consistently, as compared with corresponding normal samples.

The degree of tumour‐associated macrophage (TAM) infiltration is closely related to tumour stage and metastasis.^38^ Macrophages are the main infiltrating cells in the tumour microenvironment (TME) and play a key role in tumour growth. Macrophages participate in the immune response of tumours by polarizing into different phenotypes and polarizing into an immunosuppressive phenotype in most solid tumour. According to the theory of tumour microenvironment research, TAM can stimulate the inflammatory response by secreting pro‐inflammatory cytokines or suppress the immune response by releasing high levels of anti‐inflammatory cytokines.^47^ Tumour‐associated macrophages can be classified into M1‐phenotype and M2‐phenotype according to their functions. Condeelis et al. reported that M1‐phenotype macrophages reduce the cellular activity of human non‐small cell lung cancer cell line A549 cells by inducing apoptosis, whereas M2‐phenotype macrophage promotes tumour progression by secreting IL‐10.^48^ In this study, we found a significant positive correlation between MMP14, INHBA expression and M2‐phenotype macrophage infiltration level by bioinformatic analysis in TCGA data set. THP‐1 cell was induced to M0‐phenotype macrophage by adding PMA, and M0‐phenotype macrophage was induced to M2‐phenotype by adding MCSF. The expression levels of MMP14 and INHBA in PANC1 cells were treated with macrophage M2‐conditioned medium (MCM) and could be up‐regulated. When we analyse the correlation between the immune response and risk score based on immure‐related genes, we found CCL11, CCL13, CCL18, CCL7, CX3CL1, CXCL10, CXCL14, CXCL16, CXCL5 and CXCL8 were positively correlated with risk score, it can be laterally reflected that MMP14 and INHBA may be associated with these chemokines, and it can be speculated that M2‐phenotype macrophages upregulate the expression of MMP14 and INHBA through these chemokines.

We used multiple data set analyses to illustrate the strength of our results. However, there are various limitations in this study, which need to be further optimized. For example, some preliminary experiments are needed to expose the mechanism of INHBA and MMP14 expression in pancreatic cancer and their correlation with macrophages M2. But the prognostic model we constructed could improve clinical management.

## CONFLICT OF INTEREST

The authors declare that the research was conducted in the absence of any commercial or financial relationships that could be construed as a potential conflict of interest.

## AUTHOR CONTRIBUTIONS

**Zhan‐Wen Liang:** Data curation (equal); Investigation (equal); Methodology (equal); Writing – original draft (equal). **Jie Yu:** Data curation (equal); Investigation (equal); Writing – original draft (equal). **Dong‐Mei Gu:** Data curation (equal); Investigation (equal); Writing – original draft (equal). **Xiao‐Meng Liu:** Data curation (equal); Formal analysis (equal); Writing – review & editing (equal). **Jin Liu:** Data curation (equal); Formal analysis (equal); Writing – review & editing (equal). **Meng‐Yao Wu:** Data curation (equal); Investigation (equal); Writing – review & editing (equal). **Meng‐Dan Xu:** Data curation (equal); Investigation (equal); Writing – review & editing (equal). **Meng Shen:** Data curation (equal); Investigation (equal); Writing – review & editing (equal). **Weiming Duan:** Data curation (equal); Investigation (equal); Resources (equal); Supervision (equal). **Wei Li:** Conceptualization (equal); Data curation (equal); Investigation (equal); Project administration (equal); Writing – review & editing (equal).

## Supporting information

Table S1

Table S2

Table S3
