# Supplementary material for: M2‐phenotype tumour‐associated macrophages upregulate the expression of prognostic predictors MMP14 and INHBA in pancreatic cancer
Source: J Cell Mol Med. 2022 Feb 12;26(5):1540–55. doi: 10.1111/jcmm.17191 (PMC8899166; doi:10.1111/jcmm.17191)
Supplement: Supplementary file 1 — Table S1 [file JCMM-26-1540-s003.docx]

| **Table S1 Gene-set enrichment analysis of differentially expressed genes** | | | |
| --- | --- | --- | --- |
| **NAME** | **SIZE** | **NES** | **NOM p-val** |
| ADAPTIVE_IMMUNE_RESPONSE | 125 | -1.7120847 | 0.007692 |
| IMMUNE_RESPONSE_REGULATING_SIGNALING_PATHWAY | 180 | -1.6460576 | 0.034816 |
| NEGATIVE_REGULATION_OF_IMMUNE_SYSTEM_PROCESS | 166 | -1.5530244 | 0.034091 |
| POSITIVE_REGULATION_OF_IMMUNE_EFFECTOR_PROCESS | 68 | -1.5820951 | 0.047801 |
| POSITIVE_REGULATION_OF_IMMUNE_RESPONSE | 248 | -1.6559838 | 0.035088 |
| POSITIVE_REGULATION_OF_IMMUNE_SYSTEM_PROCESS | 358 | -1.6297832 | 0.032692 |
| REGULATION_OF_ADAPTIVE_IMMUNE_RESPONSE | 46 | -1.5695676 | 0.044944 |
| REGULATION_OF_IMMUNE_EFFECTOR_PROCESS | 126 | -1.6062707 | 0.040462 |
| REGULATION_OF_IMMUNE_RESPONSE | 318 | -1.6646392 | 0.028958 |
