# Supplementary material for: M2‐phenotype tumour‐associated macrophages upregulate the expression of prognostic predictors MMP14 and INHBA in pancreatic cancer
Source: J Cell Mol Med. 2022 Feb 12;26(5):1540–55. doi: 10.1111/jcmm.17191 (PMC8899166; doi:10.1111/jcmm.17191)
Supplement: Supplementary file 2 — Table S2 [file JCMM-26-1540-s002.docx]

| **Table S2. Immune biological process and corresponding immune genes** | |
| --- | --- |
| **Biological Process** | **Gene** |
| GO_ADAPTIVE_IMMUNE_RESPONSE | CEACAM1, GCNT3, EMP2, CD55, FOXJ1, TNFRSF21, PVR, AZGP1, IL20RB, RORC, ANXA1, PRKCZ, BCL10, GATA3, KDELR1, HPRT1, BATF, UNG, IGLL5, APCS, PARP3, RNF19B, CRP, FGL1, UNC93B1, GNL1, HPX, ERCC1, TRIM27, TRAF2, TREX1, C5, ERAP2, HLA-G, B2M, CCL19, CLCF1, MAD2L2, PRKCD, HMGB1, THOC1, C1R, IL18, CLU, RIPK2, C4A, HLA-E, C6, CTSL, SOCS5, RSAD2, TAP1, IL33, C1S, PYCARD, ORAI1, C4B, TCIRG1, CD81, JAM3, TNFSF4, IL23A, DUSP22, CXCL13, C2, HLA-DRB1, IL6ST, CD74, LAT, CR2, CD79A, PRDM1, RORA, FOXP3, HLA-DQB1, RELB, SLAMF7, CTSS, LEF1, CD7, HLA-DRA, HLA-DRB5, HLA-DQA1, HLA-DOB, SWAP70, TNFSF13B, CD27, SLAMF6, CD79B, LYN, IL7R, ICAM1, CD1C, FGL2, HLA-DMA, CD3D, GPR183, TNFRSF1B, SYK, RFTN1, CD3E, SIT1, FCER1G, HLA-DPA1, TLR4, C1QA, HLA-DPB1, HAVCR2, HLA-DQB2, SAMSN1, C1QC, C1QB, JAK3, PTPRC, CD48, HLA-DOA, IL27RA, HLA-DMB, CD86, CD4, PRF1, WAS, SPN, HLA-DQA2, SASH3 |
| GO_IMMUNE_RESPONSE_REGULATING_SIGNALING_PATHWAY | MUC13, TRIM15, KCNN4, CEACAM1, DMBT1, S100A14, MUC5AC, MUC5B, F2RL1, MUC3A, MUC17, TNFRSF21, MUC4, IL20RB, PIGR, MUC6, LGALS3, BPIFB1, FOS, MAPK3, HHLA2, WASL, COLEC12, LGR4, BCL10, EZR, HSP90AA1, GATA3, PPP3CA, TICAM1, PSMD1, FCER1A, DDX60, RAC1, IRAK2, MAP2K6, UBE2V1, PSMC1, PAK3, PLEKHA1, IGLL5, PSMC4, HSP90AB1, CARD11, PELI1, LRRC14, THY1, UNC93B1, PSMA6, SKP1, EIF2B4, PSMC6, PSMB3, CRKL, RPS3, PSMD6, CBFB, PSMA2, UBE2D3, PSMD9, TICAM2, PIK3R2, PRKACB, CUL1, EIF2B5, RPS6KA3, CRK, ACTB, RPS27A, PDPK1, ACTG1, HLA-G, BRK1, WDFY1, CTSK, RAF1, PRKCD, PAK2, HMGB1, UBA52, MAPKAPK2, PSME1, PRNP, RAB7B, CDC42, RUNX1, TYRO3, ZCCHC3, PTPN2, ABI1, PLA2G6, RNF31, IKBKG, RIPK2, PSMB8, PTPRJ, MYD88, MFHAS1, SARM1, IKBKB, NRAS, PVRIG, CTSL, CAV1, RAB29, FCGR2A, RSAD2, ARPC1B, CTSB, PSMB10, PIK3C3, MARCO, CMTM3, ACTR2, LGMN, REG3G, CD81, GRB2, BIRC3, LCK, PSMB9, GFI1, DDX58, GPS2, DUSP22, HLA-DRB1, CD14, NFATC2, LAT, CR2, C5AR1, CD79A, TIFA, S100A9, FOXP3, HLA-DQB1, RELB, CTSS, HLA-DRA, BTNL9, FPR3, HLA-DRB5, HLA-DQA1, GBP1, ICAM3, CD79B, LYN, CCR7, ITGAM, IKBKE, VAV1, C3AR1, LY96, CD3D, TRAF3, SYK, TLR2, RFTN1, CD3E, WIPF1, FCER1G, HLA-DPA1, TLR4, CD36, HLA-DPB1, FPR1, HAVCR2, FCGR3A, ITGB2, HLA-DQB2, PTPRC, MNDA, CD4, THEMIS2, CD300LF, WAS, HLA-DQA2, NFAM1, HCK, CD300A |
| GO_NEGATIVE_REGULATION_OF_IMMUNE_SYSTEM_PROCESS | IHH, CEACAM1, MMP28, CLDN18, HOXA5, SHH, F2RL1, BMP4, CD55, PPARG, FOXJ1, TNFRSF21, IL20RB, SOX9, KITLG, FOXF1, LGALS3, BPIFB1, NBL1, HES1, LDLR, TRIB1, ANXA1, SOCS6, MDK, ADTRP, WASL, GREM1, LEO1, NME1, LGR4, BCL10, EZR, CDC73, TICAM1, FAM3A, TMEM176A, GPR68, ZFP36, SDC4, TOB2, INHBA, CEBPB, MIF, APCS, MIA3, PARP3, PELI1, LRRC14, PAF1, THY1, FGL1, COL3A1, TMEM176B, MUL1, WDR61, NME2, FBN1, RNF26, DDT, TRIM27, RPS19, CBFB, TWSG1, IDO1, CTNNB1, TICAM2, TREX1, AKT1, INS, CASP3, C5, TARBP2, CRK, PDPK1, GATA2, HLA-G, ADAR, RARA, ZC3H8, NMI, DUSP1, PCBP2, HMGB1, CX3CL1, N4BP2L2, LRRC32, AXL, PRNP, RAB7B, THOC1, BST2, FBXW7, SYT11, RUNX1, TYRO3, PTPN2, HTRA1, LRCH1, PTPRJ, MFHAS1, SARM1, GPX1, AMBP, HLA-E, THBS1, PVRIG, SOCS5, ZBTB16, VSIG4, IL33, IL2RA, METTL3, GRN, MEIS1, SFRP1, PARP14, TNFSF4, IFI16, TRAFD1, GPS2, DUSP22, EMILIN1, CD14, CD74, MAFB, CXCL12, A2M, GPNMB, FOXP3, ATM, SOCS1, CCL21, MILR1, UBASH3B, CST7, SERPINB9, HLA-DOB, GBP1, LYN, IL7R, CD68, FGL2, TRIM38, LY96, FCER1G, RUNX3, TLR4, TYROBP, SLAMF8, HAVCR2, SAMSN1, C1QC, JAK3, PTPRC, MNDA, HLA-DOA, IL27RA, CD86, LST1, CD300LF, SPN, MMP12, TNFAIP8L2, CD300A, HMOX1 |
| GO_POSITIVE_REGULATION_OF_IMMUNE_EFFECTOR_PROCESS | TRIM15, KLK7, F2RL1, ADORA2B, PVR, AZGP1, FOXF1, ANXA1, PRKCZ, MAPK3, BCL10, GATA3, TICAM1, DDX60, MIF, HPX, HK1, RPS19, SNX4, XBP1, TRAF2, DHX36, PDPK1, GATA2, HLA-G, NPPA, RARA, B2M, CCL19, CLCF1, MAD2L2, VAMP8, ZCCHC3, MZB1, IL18, RIPK2, PTPRJ, STXBP2, HLA-E, C6, SOCS5, RSAD2, IL33, CD81, TNFSF4, IL23A, DDX58, CD74, FOXP3, SEMA7A, RAC2, PTAFR, SLAMF6, LYN, ITGAM, VAV1, CD1C, SYK, FCER1G, TLR4, CD36, ITGB2, PTPRC, HLA-DMB, CD86, SASH3, CD300A, HMOX1 |
| GO_POSITIVE_REGULATION_OF_IMMUNE_RESPONSE | MUC13, TRIM15, KCNN4, CEACAM1, DMBT1, KLK7, S100A14, MUC5AC, MUC5B, F2RL1, ADORA2B, CD55, MUC3A, MUC17, TNFRSF21, PVR, MUC4, AZGP1, MUC6, LGALS3, BPIFB1, ANXA1, CFH, PRKCZ, MAPK3, HHLA2, WASL, COLEC12, LGR4, BCL10, EZR, HSP90AA1, ADAM8, GATA3, TICAM1, PSMD1, DDX60, RAC1, IRAK2, MAP2K6, UBE2V1, PSMC1, PAK3, PLEKHA1, IGLL5, MIF, PSMC4, APCS, HSP90AB1, CARD11, PELI1, LRRC14, THY1, CRP, UNC93B1, PSMA6, HPX, SKP1, EIF2B4, PSMC6, HEXIM1, HK1, PROS1, RPS19, SNX4, PSMB3, CRKL, RPS3, PSMD6, CBFB, PSMA2, RGCC, IDO1, XBP1, UBE2D3, PSMD9, TRAF2, TICAM2, TREX1, PIK3R2, DHX36, PRKACB, CUL1, C5, EIF2B5, RPS6KA3, CRK, ACTB, RPS27A, PDPK1, GATA2, ACTG1, HLA-G, NPPA, RARA, BRK1, HMGB2, WDFY1, B2M, CTSK, COCH, RAF1, CCL19, CLCF1, MAD2L2, PRKCD, PAK2, HMGB1, UBA52, MAPKAPK2, FCN3, PSME1, PRNP, RAB7B, MATR3, CDC42, VAMP8, PLA2G1B, RUNX1, TYRO3, ZCCHC3, C1R, PTPN2, SFPQ, IL18, ABI1, CLU, PLA2G6, RNF31, IKBKG, RIPK2, PSMB8, PTPRJ, MYD88, MFHAS1, SARM1, C4A, IKBKB, NRAS, HLA-E, C6, PVRIG, CTSL, CAV1, SOCS5, VSIG4, CFB, RAB29, FCGR2A, RSAD2, ARPC1B, CTSB, PSMB10, PIK3C3, MARCO, IL33, C1S, PYCARD, CMTM3, ACTR2, LGMN, C4B, REG3G, CD81, GRB2, BIRC3, LCK, PSMB9, TNFSF4, IL23A, IFI16, GFI1, DDX58, GPS2, DUSP22, C2, HLA-DRB1, IL6ST, CD14, CD74, NFATC2, LAT, CR2, C5AR1, CD79A, A2M, TIFA, S100A9, FOXP3, HLA-DQB1, RELB, SEMA7A, CTSS, HLA-DRA, BTNL9, FPR3, HLA-DRB5, HLA-DQA1, PTAFR, TNFSF13B, GBP1, SLAMF6, ICAM3, CD79B, LYN, CCR7, ITGAM, IKBKE, VAV1, CD1C, C3AR1, LY96, CD3D, TRAF3, CCL5, SYK, TLR2, RFTN1, CD3E, WIPF1, FCER1G, HLA-DPA1, TLR4, C1QA, CD36, HLA-DPB1, FPR1, HAVCR2, FCGR3A, ITGB2, HLA-DQB2, C1QC, C1QB, PTPRC, MNDA, IL27RA, HLA-DMB, CD86, CD4, THEMIS2, CD300LF, WAS, HLA-DQA2, SASH3, NFAM1, HCK, MMP12, CD300A |
| GO_POSITIVE_REGULATION_OF_IMMUNE_SYSTEM_PROCESS | MUC13, TRIM15, IHH, KCNN4, CEACAM1, DMBT1, KLK7, S100A14, MUC5AC, MUC5B, HOXA5, SHH, F2RL1, ADORA2B, EFNB2, CD55, FOXJ1, MUC3A, MUC17, LIF, SLC9B2, TNFRSF21, PVR, MUC4, AZGP1, MUC6, FOXC1, ITGA2, KITLG, FOXF1, LGALS3, STK39, BPIFB1, EFNB1, HES1, TRIB1, FOS, ANXA1, CFH, DPP4, CCL20, MDK, PRKCZ, MAPK3, HHLA2, WASL, COLEC12, THBS4, LGR4, BCL10, FAM210B, EZR, HSP90AA1, ADAM8, GATA3, IGF2, TICAM1, TNFSF9, CCL24, PSMD1, DDX60, SERPINE1, RAC1, IRAK2, GPR68, MAP2K6, PNP, UBE2V1, PSMC1, PAK3, INHBA, PLEKHA1, IGLL5, SPACA3, MIF, PSMC4, APCS, GLI2, GNAS, MIA3, VEGFA, HSP90AB1, IRS2, CARD11, PELI1, LRRC14, THY1, CRP, UNC93B1, PSMA6, CALR, HPX, ANO6, SKP1, EIF2B4, PSMC6, PCID2, HEXIM1, HK1, PROS1, SMAP1, CD99, ROR2, TNFSF11, BLOC1S3, ISG15, RPS19, SNX4, PSMB3, CRKL, RPS3, PSMD6, CBFB, VEGFC, PSMA2, APP, RGCC, IDO1, BST1, PTPN11, AKIRIN1, XBP1, UBE2D3, PSMD9, TRAF2, TICAM2, TREX1, PIK3R2, AKT1, DHX36, PRKACB, TGFBR2, CUL1, C5, EIF2B5, RPS6KA3, CRK, ACTB, SELP, RPS27A, PDPK1, GATA2, ACTG1, HLA-G, NPPA, RARA, SH3KBP1, BRK1, HMGB2, WDFY1, B2M, CTSK, VNN1, COCH, MMP14, RAF1, PDCD2, CCL19, CLCF1, MAD2L2, PRKCD, PAK2, HMGB1, CX3CL1, RARRES2, N4BP2L2, UBA52, AXL, MAPKAPK2, FCN3, PSME1, PRNP, RAB7B, RHOA, CXCL8, MATR3, CDC42, VAMP8, LGALS1, ACIN1, PLA2G1B, RUNX1, TYRO3, ZCCHC3, C1R, MZB1, PTPN2, SFPQ, IL18, ABI1, CLU, EPO, PLA2G6, RNF31, IKBKG, FLOT2, RIPK2, IGFBP2, PSMB8, PTPRJ, STXBP2, MYD88, MFHAS1, SARM1, P2RX4, C4A, IKBKB, NRAS, HLA-E, RB1, THBS1, C6, PVRIG, CTSL, CAV1, SOCS5, ZBTB16, VSIG4, PLVAP, CXCL14, CFB, RAB29, FCGR2A, RSAD2, ARPC1B, CTSB, PSMB10, PIK3C3, MARCO, IL33, C1S, IL2RA, PYCARD, CMTM3, STAT1, ACTR2, EDN1, LGMN, C4B, REG3G, CD81, HIF1A, EGR3, GRB2, BIRC3, LCK, PSMB9, TNFSF4, IL23A, IFI16, GFI1, TNFRSF18, DDX58, GPS2, PTN, DUSP22, CXCL13, C2, HLA-DRB1, IL6ST, TREM2, CD14, CD74, NFATC2, CXCL10, LAT, CXCL12, CR2, C5AR1, CD79A, A2M, TIFA, CD5, S100A9, FOXP3, HLA-DQB1, RELB, SEMA7A, CTSS, LEF1, SOCS1, HLA-DRA, BTNL9, CCL21, FPR3, HLA-DRB5, HLA-DQA1, SIRPA, RAC2, PTAFR, SWAP70, TNFSF13B, CD27, GBP1, SLAMF6, ICAM3, CD79B, LYN, CCR7, ITGAM, IL7R, IKBKE, VAV1, ICAM1, CD1C, C3AR1, ETS1, LY96, CD3D, TRAF3, GPR183, CCL5, SYK, TLR2, RFTN1, CD3E, WIPF1, VCAM1, FCER1G, CD2, HLA-DPA1, RUNX3, TLR4, TYROBP, C1QA, CD36, HLA-DPB1, FPR1, GPSM3, HAVCR2, FCGR3A, ITGB2, CD83, PLA2G7, HLA-DQB2, C1QC, C1QB, JAK3, PTPRC, MNDA, IL27RA, CCR1, HLA-DMB, AIF1, CD86, CD4, THEMIS2, EVI2B, CD300LF, WAS, SPN, HLA-DQA2, SASH3, NFAM1, HCK, MMP12, CD300A, HMOX1 |
| GO_REGULATION_OF_ADAPTIVE_IMMUNE_RESPONSE | CEACAM1, FOXJ1, PVR, AZGP1, IL20RB, ANXA1, PRKCZ, GATA3, PARP3, HPX, TRIM27, TRAF2, HLA-G, B2M, CCL19, CLCF1, MAD2L2, HMGB1, THOC1, IL18, RIPK2, HLA-E, SOCS5, RSAD2, IL33, PYCARD, CD81, TNFSF4, IL23A, DUSP22, IL6ST, FOXP3, TNFSF13B, IL7R, CD1C, TNFRSF1B, FCER1G, HAVCR2, SAMSN1, JAK3, PTPRC, CD48, IL27RA, CD4, WAS, SASH3 |
| GO_REGULATION_OF_IMMUNE_EFFECTOR_PROCESS | TRIM15, CEACAM1, KLK7, F2RL1, ADORA2B, CD55, FOXJ1, PVR, AZGP1, IL20RB, FOXF1, LGALS3, ANXA1, CFH, PRKCZ, MAPK3, BCL10, GATA3, TICAM1, ZMPSTE24, DDX60, MIF, PARP3, HPX, MUL1, DNASE1, HK1, PROS1, RNF26, RPS19, SNX4, IFIT1, XBP1, TRAF2, TREX1, INS, DHX36, C5, TARBP2, CRK, PDPK1, GATA2, HLA-G, NPPA, DTX3L, RARA, B2M, CCL19, CLCF1, MAD2L2, PCBP2, HMGB1, VAMP8, THOC1, BST2, ZCCHC3, C1R, MZB1, IL18, HTRA1, CLU, RIPK2, PTPRJ, STXBP2, C4A, HLA-E, C6, SOCS5, VSIG4, CFB, RSAD2, IL33, C1S, IL2RA, PYCARD, STAT1, C4B, CD81, GRN, BIRC3, TNFSF4, IL23A, DDX58, DUSP22, C2, CD74, CR2, C5AR1, A2M, FOXP3, SEMA7A, SERPINB9, RAC2, PTAFR, SLAMF6, LYN, ITGAM, IL7R, VAV1, CD1C, FGL2, C3AR1, TRIM38, TRAF3, TNFRSF1B, SYK, FCER1G, TLR4, SLAMF8, C1QA, CD36, HAVCR2, ITGB2, C1QC, C1QB, JAK3, PTPRC, IL27RA, HLA-DMB, CD86, CXCL6, WAS, SASH3, MMP12, CD300A, HMOX1 |
| GO_REGULATION_OF_IMMUNE_RESPONSE | MUC13, COL17A1, TRIM15, KCNN4, CEACAM1, DMBT1, KLK7, S100A14, MUC5AC, MUC5B, F2RL1, ADORA2B, CD55, PPARG, FOXJ1, MUC3A, MUC17, TNFRSF21, PVR, MUC4, AZGP1, IL20RB, PIGR, MUC6, FOXF1, LGALS3, BPIFB1, ECM1, FOS, ANXA1, CFH, PRKCZ, MAPK3, HHLA2, WASL, COLEC12, LGR4, BCL10, EZR, HSP90AA1, ADAM8, GATA3, PPP3CA, TICAM1, PSMD1, FCER1A, DDX60, RAC1, FAM3A, IRAK2, MAP2K6, UBE2V1, PSMC1, PAK3, PLEKHA1, IGLL5, PILRB, COL1A1, MIF, PSMC4, APCS, SOCS3, SUMO1, HSP90AB1, CARD11, PARP3, COL1A2, PELI1, LRRC14, THY1, CRP, FGL1, UNC93B1, COL3A1, PSMA6, HPX, MUL1, SKP1, EIF2B4, PSMC6, HEXIM1, HK1, PROS1, CD99, TRIM27, RPS19, SNX4, PSMB3, CRKL, RPS3, PSMD6, CBFB, PSMA2, RGCC, IDO1, PTPN11, XBP1, UBE2D3, PSMD9, TRAF2, IFITM1, TICAM2, TREX1, PIK3R2, INS, DHX36, PRKACB, CUL1, C5, EIF2B5, RPS6KA3, CRK, ACTB, RPS27A, PDPK1, GATA2, ACTG1, HLA-G, NPPA, ADAR, RARA, BRK1, HMGB2, WDFY1, SPPL2B, B2M, CTSK, IFNGR2, COCH, ITGB1, RAF1, CCL19, CLCF1, NMI, MAD2L2, PRKCD, PAK2, HMGB1, UBA52, MAPKAPK2, FCN3, PSME1, PRNP, RAB7B, MATR3, CDC42, SPPL2A, VAMP8, THOC1, PLA2G1B, BST2, RUNX1, TYRO3, ZCCHC3, C1R, PTPN2, SFPQ, IL18, ABI1, CLU, DNASE2, PLA2G6, RNF31, IKBKG, RIPK2, PSMB8, PTPRJ, STXBP2, MYD88, MFHAS1, SARM1, C4A, GPX1, TREM1, IKBKB, AMBP, PTPN1, NRAS, HLA-E, C6, PVRIG, CTSL, CAV1, SOCS5, VSIG4, CFB, RAB29, FCGR2A, RSAD2, ARPC1B, CTSB, PSMB10, PIK3C3, MARCO, IL33, C1S, IL2RA, PYCARD, METTL3, CMTM3, STAT1, ACTR2, LGMN, C4B, REG3G, CD81, GRN, JAK1, PARP14, GRB2, BIRC3, LCK, PSMB9, TNFSF4, IL23A, IFI16, GFI1, TRAFD1, DDX58, GPS2, DUSP22, CXCL13, C2, HLA-DRB1, IL6ST, TREM2, CD14, IFNGR1, CD74, NFATC2, LAT, CR2, C5AR1, CD79A, A2M, TIFA, S100A9, FOXP3, HLA-DQB1, RELB, SEMA7A, SLAMF7, CTSS, SOCS1, HLA-DRA, BTNL9, CLEC2B, FPR3, HLA-DRB5, HLA-DQA1, SERPINB9, RAC2, PTAFR, TNFSF13B, GBP1, SLAMF6, ICAM3, CD79B, LYN, CCR7, ITGAM, IL7R, IKBKE, SELL, VAV1, ICAM1, CD1C, FGL2, C3AR1, LY96, CD3D, TRAF3, CCL5, OSCAR, TNFRSF1B, SYK, TLR2, RFTN1, CD3E, WIPF1, VCAM1, FCER1G, HLA-DPA1, TLR4, TYROBP, SLAMF8, C1QA, CD36, HLA-DPB1, FPR1, HAVCR2, FCGR3A, ITGB2, HLA-DQB2, SAMSN1, C1QC, C1QB, JAK3, PTPRC, APOE, MNDA, CD48, IL27RA, HCST, HLA-DMB, KLRB1, CD86, CD4, THEMIS2, CD300LF, WAS, HLA-DQA2, SASH3, NFAM1, HCK, MMP12, CD300A, HMOX1 |
