# Supplementary material for: M2‐phenotype tumour‐associated macrophages upregulate the expression of prognostic predictors MMP14 and INHBA in pancreatic cancer
Source: J Cell Mol Med. 2022 Feb 12;26(5):1540–55. doi: 10.1111/jcmm.17191 (PMC8899166; doi:10.1111/jcmm.17191)
Supplement: Supplementary file 3 — Table S3 [file JCMM-26-1540-s001.docx]

| **Table S3. Univariate Cox regression analysis of immune genes.** | | |
| --- | --- | --- |
| **Gene** | **Hazard Ratio** | **pvalue** |
| IGFBP2 | 0.795221492 | 0.005331322 |
| NRAS | 2.205398356 | 0.000266259 |
| IRAK2 | 1.607532473 | 7.07601E-05 |
| DNASE1 | 0.477283326 | 0.003836283 |
| PPP3CA | 1.991912701 | 0.001463391 |
| DTX3L | 2.25539086 | 0.00026491 |
| CEBPB | 1.378704426 | 0.005506751 |
| TAP1 | 1.60894683 | 0.000433119 |
| SOX9 | 1.379074813 | 0.005991563 |
| PSME1 | 1.868039247 | 0.006477691 |
| FOXC1 | 1.505676671 | 0.003050508 |
| SFRP1 | 0.813950013 | 0.007059139 |
| DDX60 | 1.555392817 | 0.000151748 |
| IL20RB | 1.406545786 | 3.9335E-06 |
| EFNB2 | 1.767668923 | 7.41272E-05 |
| LDLR | 1.399503765 | 0.000544847 |
| INHBA | 1.276901492 | 0.004260206 |
| SPPL2B | 0.643666991 | 0.000258981 |
| CAV1 | 1.329510648 | 0.002598423 |
| KITLG | 1.375347885 | 0.00628043 |
| SH3KBP1 | 1.69154715 | 0.007441577 |
| TNFRSF21 | 1.590267739 | 0.00170386 |
| RUNX1 | 1.588504208 | 0.006692768 |
| NME2 | 2.638942987 | 0.000287936 |
| PSMB9 | 1.640076119 | 0.000189429 |
| FOXJ1 | 0.811109123 | 0.007569682 |
| GBP1 | 1.455532273 | 0.001535922 |
| ANO6 | 1.898446562 | 0.000313548 |
| RIPK2 | 2.080343554 | 1.74823E-05 |
| PPARG | 1.305111022 | 0.00263163 |
| ITGA2 | 1.522813931 | 0.000142324 |
| PSMA6 | 2.703438647 | 0.002131438 |
| ACTR2 | 1.735675953 | 0.005557031 |
| MMP14 | 1.352609159 | 0.002182134 |
| LRRC14 | 0.464033977 | 0.001351411 |
| IFITM1 | 1.292906576 | 0.004413899 |
| TMEM176A | 0.752018555 | 0.008144407 |
| MYD88 | 1.652650966 | 0.00512708 |
| BIRC3 | 1.334421839 | 0.001355978 |
| EFNB1 | 1.507670188 | 0.000362721 |
| NPPA | 0.091953559 | 0.001361982 |
| ECM1 | 1.231084748 | 0.007682558 |
| TMEM176B | 0.755530373 | 0.008548589 |
| RSAD2 | 1.370048822 | 0.005308549 |
| BCL10 | 1.499442946 | 0.004503014 |
| GNAS | 0.619561597 | 0.004207579 |
| PARP14 | 1.665301243 | 0.001133269 |
| ANXA1 | 1.429071338 | 0.000238861 |
| IFI16 | 1.452213041 | 0.003593995 |
| HMGB2 | 1.575301635 | 0.00549194 |
| UBASH3B | 1.931115654 | 0.000164162 |
| CLEC2B | 1.392646291 | 0.005591092 |
| MMP28 | 1.509693001 | 1.19747E-05 |
| CLCF1 | 1.359796036 | 0.004522823 |
| KCNN4 | 1.320957056 | 0.000212108 |
| COL17A1 | 1.27890326 | 1.44387E-05 |
| S100A14 | 1.229628118 | 0.000465322 |
| BTNL9 | 0.527048558 | 0.000659042 |
| EZR | 1.551549291 | 0.000453461 |
| GNL1 | 0.40590013 | 0.00182156 |
